# A Comprehensive Review of the Composition, Nutritional Value, and Functional Properties of Camel Milk Fat

**DOI:** 10.3390/foods10092158

**Published:** 2021-09-13

**Authors:** Ibrahim A. Bakry, Lan Yang, Mohamed A. Farag, Sameh A. Korma, Ibrahim Khalifa, Ilaria Cacciotti, Noha I. Ziedan, Jun Jin, Qingzhe Jin, Wei Wei, Xingguo Wang

**Affiliations:** 1 Collaborative Innovation Center of Food Safety and Quality Control in Jiangsu Province, School of Food Science and Technology, Jiangnan University, Wuxi 214122, China; ibrahimbakry@zu.edu.eg (I.A.B.); wangxg1002@gmail.com (J.J.); jqzwuxi@163.com (Q.J.); weiw@jiangnan.edu.cn (W.W.); 2 Department of Food and Dairy Technology, Faculty of Technology and Development, Zagazig University, Zagazig 44519, Egypt; 3 Key Laboratory of Dairy Biotechnology and Engineering, Ministry of Education, Inner Mongolia Agricultural University, Huhhot 010018, China; sunnylan07@126.com; 4 Pharmacognosy Department, College of Pharmacy, Cairo University, Kasr el Aini St., Cairo 11562, Egypt; mohamed.alifarag@aucegypt.edu; 5 Department of Food Science, Faculty of Agriculture, Zagazig University, Zagazig 44519, Egypt; sameh.hosny@zu.edu.eg; 6 Food Technology Department, Faculty of Agriculture, Benha University, Moshtohor 13734, Egypt; Ibrahiem.khalifa@fagr.bu.edu.eg; 7 Department of Engineering, INSTM RU, University of Rome “Niccolò Cusano”, 00166 Roma, Italy; ilaria.cacciotti@unicusano.it; 8 Department of Mathematical and Physical Sciences, Faculty of Science and Engineering, University of Chester, Thornton Science Park, Pool Ln, Chester CH2 4NU, UK; n.ziedan@chester.ac.uk

**Keywords:** camel milk, milk fat globules, cholesterol, fatty acids, phospholipids, health-promoting benefits

## Abstract

Recently, camel milk (CM) has been considered as a health-promoting icon due to its medicinal and nutritional benefits. CM fat globule membrane has numerous health-promoting properties, such as anti-adhesion and anti-bacterial properties, which are suitable for people who are allergic to cow’s milk. CM contains milk fat globules with a small size, which accounts for their rapid digestion. Moreover, it also comprises lower amounts of cholesterol and saturated fatty acids concurrent with higher levels of essential fatty acids than cow milk, with an improved lipid profile manifested by reducing cholesterol levels in the blood. In addition, it is rich in phospholipids, especially plasmalogens and sphingomyelin, suggesting that CM fat may meet the daily nutritional requirements of adults and infants. Thus, CM and its dairy products have become more attractive for consumers. In view of this, we performed a comprehensive review of CM fat’s composition and nutritional properties. The overall goal is to increase knowledge related to CM fat characteristics and modify its unfavorable perception. Future studies are expected to be directed toward a better understanding of CM fat, which appears to be promising in the design and formulation of new products with significant health-promoting benefits.

## 1. Introduction

Understanding the relationship between components of milk fats, diet, and health is now known to be one of the key concepts to a better lifestyle, disease prevention, and well-being promotion. Milk consumption for all mammals except cows has increased by 17% in the last 50 years in all countries [1]. Camels are a cultural, economic, and health-promoting icon [2] with an estimated worldwide population of over 35 million [3]. There are two species of camels: one-humped camels (*Camelus dromedarius*) and two-humped camels (*Camelus bactrianus*) [4]. Camel milk (CM) is an essential food source in arid and semi-arid areas, as cow milk is not always available in these areas due to a lack of adaptation mechanisms. Despite these conditions, CM has been considered as one of the best alternatives to feed adults and infants [5,6], as well as for the production of many dairy products such as yogurt, cheese, cream, and butter [7]. Besides its nutritive value, CM has also long been recognized for its health benefits by nomadic people for centuries, and recent studies have revealed its potential value in the treatment of a variety of human diseases such as asthma, tuberculosis, jaundice, and gastrointestinal diseases [8]. However, despite these interesting properties and applications, it has not received as much attention as cow milk.

**Figure 1:**
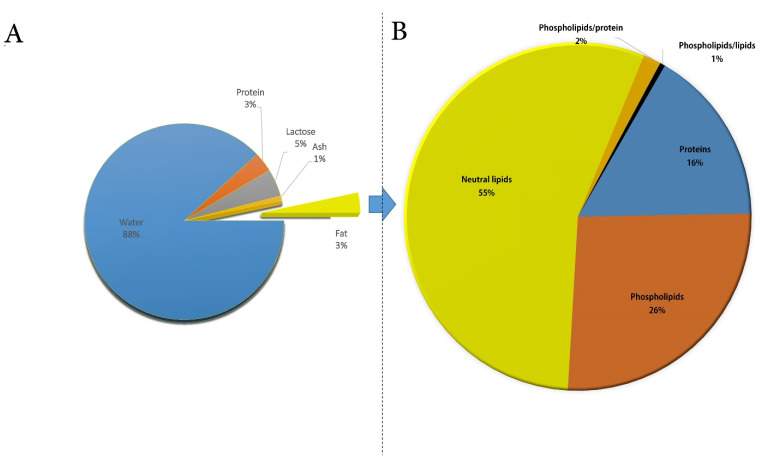
(**A**) Chemical composition of camel milk. (**B**) Composition of milk fat globule membrane in camel milk. The charts were created considering the data reported in Ref. [9] (**A**) and [29] (**B**).

Reported data collected from 121 references published between 1905 and 2019 showed that the mean and standard deviation of components in CM were 12.2 ± 1.62% total solids, 3.28 ± 0.59 protein, 4.47 ± 0.66 lactose, 0.81 ± 0.19 ash, and 3.68 ± 1.00 fat (Figure 1A) [9]. CM fat exists as milk fat globules (MFGs) in the water, with a size ranging from 1.1 to 2.1 mm, which is lower than those of buffalo (3.9–7.7 mm), cow (1.6–4.9 mm), and goat milk (1.1–3.9 mm) [10], justifying its faster digestion rate in comparison to other mammalian milk. MFGs are covered with a layer of a surface-active substance called milk fat globule membrane (MFGM), and they have many nutritional and health roles. Similar to other milk types, the composition of CM fat constantly changes as a result of many environmental and physiological influences [11,12,13]. Such chemical changes affect its physical properties and its dairy products [12,14]. Fat is an important component of CM, including a complex mixture of natural fats (i.e., triglycerides, phospholipids, cholesterol, and other elements), representing one of the sources of energy, in addition to fat-soluble vitamins. Moreover, CM fat is an excellent source of essential fatty acids (EFAs) [12,15] and may meet the daily nutritional requirements of countries whose traditional diet is high in carbohydrates. Indeed, the consumption of human milk in these countries commonly leads to a low level of EFAs, such as alpha-linolenic acid (ALA) and linoleic acid (LA) [16,17]. Another characteristic lipid profile of CM includes its good source of polyunsaturated FAs (PUFAs) i.e., ALA, eicosapentaenoic acid (EPA), and arachidonic acid (AA) [18,19,20], compared to other mammalian species of milk, posing it as a better fat source for individuals at risk of lipid-related cardiovascular diseases [21,22]. It should be noted that CM cholesterol level is contradictory if compared to cow milk [11,14,23,24]. Despite some contradictory reports, several researchers have demonstrated that CM (both fresh and fermented milk) reduced the development of hypercholesterolemia in rats [25,26,27,28].

CM fat was reported to act as an emulsifier more than human or cow milk because it contains almost 1% of its total lipids as phospholipids (PLs) [6]. Furthermore, it was reported that the amount of plasmalogen and sphingomyelin (SM) were similar in camel and human milk [6]. For this reason, it can be useful in newborn nutrition if the PLs are purified and concentrated and used to complement human milk fortifiers or produce novel milk replacements.

On the basis of the aforementioned information, it is evident that the CM fat components are quite comparable to human and cow milk. Nevertheless, the majority of studies have focused on the fat composition of cow and human milk, with less reported regarding CM. The objective of the present review is to highlight the reports currently available on the detailed composition and nutritional value of CM fat for improved utilization in dairy industries with focus on the recent findings of its applications.

## 2. The Health-Promoting Properties of CM Fat

Over the last 150 years, the diet of Western societies has witnessed changes, being shifted towards a high intake of saturated FAs (SFAs) with low amounts of *n*-3 and *n*-6 FAs due to the consumption of modern fast and processed food [30,31]. Furthermore, the hydrogenation technique has permitted vegetable oils to solidify and be marketed as shortening or margarine, and therein it is being used in foods [32]. During the same period, cow milk and its products have been widely investigated. On average, cow milk contains 70% SFAs, 25% monounsaturated fatty acids (MUFAs), and 5% polyunsaturated fatty acids (PUFAs) [33], whereas the ideal FAs profile, from a human health perspective, should be 8% SFAs, 82% MUFAs, and 10% PUFAs [34]. As a result, milk fat (butter) has been criticized for its unfavorable FA profile. However, CM, especially its fat component, has received little attention. In addition, CM has a long history of negative perception, with the belief that it is low in fat and has high levels of cholesterol.

**Figure 2:**
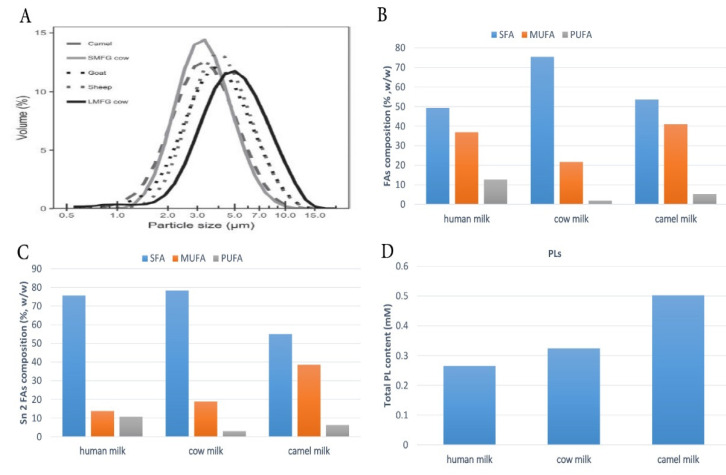
(**A**) Milk fat globule size distributions. Reprinted with permission from Ref. [45]. Copyright 2020 Elsevier; (**B**) composition of total fatty acids; (**C**) *sn*-2 fatty acids; (**D**) phospholipids (%) in human, cow, and camel milk fat. The histograms have been prepared using the data reported in [20]. Saturated fatty acids (SFAs), monounsaturated fatty acids (MUFAs), polyunsaturated fatty acids (PUFAs), Phospholipids (PLs).

In recent years, a large amount of consideration has been devoted to the design of low-saturated and high-unsaturated (UFAs) dairy products with a high level of bioactive components. In addition, considerable progress has been made in identifying the broad array of components found in milk fat. Thus, numerous research on CM fat and its impact on human health have been done. A positive relationship was found between CM and human health, leading to a drastic change in the recognition of the fat components of CM and considering it as a healthy food. CM fat differs from other mammalian milk fats due to its globule’s small size, which guarantees its easy digestion by humans (Figure 2A). In addition to its lower SFA content, CM has a higher level of MUFAs and PUFAs compared to cow milk (Figure 2B). The long-chain FAs (LC-FAs) and UFAs in CM are the most abundant, giving it a special ability to reduce the incidence of fat-related cardiovascular diseases by 35–50% [21]. Interestingly, CM fat is richer in conjugated linoleic acid (CLA) compared to human milk [35,36]. CLA has been recognized for its benefits in lowering blood glucose levels and is believed to prevent osteoporosis, enhance fat metabolism, and activate the immune system [37]. Furthermore, it is said to prevent the occurrence and progression of cancers of the stomach, colon, breast, and skin. In addition, the isomers of CLA are known to play a role in preventing obesity [38]. The distribution of three main FAs in CM fat differs considerably between human milk fat and cow milk fat (Figure 2C). CM fat has a lower SFA level than human and cow milk fat but a higher MUFA concentration, while the amount of PUFAs linked to the *sn*-2 position differs between CM fat (6.27%), cow milk fat (3.02%), and human milk fat (10.64%) (Figure 2C). In comparison to human and cow milk fat globules, CM fat globules contain a significant quantity of PLs (Figure 2D). In agro-food or clinical nutrition, PLs are one of the most essential functional lipid components [39]. Another advantage is that CM has a lower overall cholesterol level (5.64–3.18 mg/100 g) than cow milk (8.51–9.07 mg/100 g). In addition, this content fits well within the recent vision of the dairy industry, which is interested in designing and formulating products that integrate certain bioactive components obtained from milk. Generally, CM fat is considered to have many medical properties as it has been identified for its anti-diabetic [40], antibacterial [41], antiviral [42], anti-inflammatory [43], antihypertensive [44], and hypoallergenic properties [21].

## 3. Composition and Functional Properties of CM Fat Globule and Membrane

Milk is a lipid-in-water emulsion that plays an important role in human nutrition. MFGs are a combination of proteins and lipids with nutraceutical characteristics linked to the MFGM, which preserves them and prevents coalescence.

### 3.1. CM Fat Globule Membrane

The complex MFGM architecture ensures stable dispersion of MFGs in milk through polar lipids and glycoproteins present in the membrane, inducing electrostatic and steric repulsion, in addition to its nutritional and health effects inside the body. According to Lindmark Månsson [46], fats account for 30% of the membrane and can be divided into three types (i.e., phospholipids (25%), cerebrosides (3%), and cholesterol (2%)), while proteins make up the remaining 70% of the membrane. Recently, increasing attention has been paid to the components of MFGM, especially to their protein components [47]. MFGM proteins, which account for 1–4% of the total milk protein, differ depending on the breed [48]. The proteins of camel MFGM are mainly involved in protein processing, bio-synthesis of fat, and actin cytoskeleton organization [47,49]. The main MFGM proteins are FAs synthase, xanthine oxidase, butyrophilin, lactadherin, and adipophilin [49]. During the secretion of the MFGs [50], the localization of the proteins differs: some are related to the inner monolayer membrane, while others to the outer bilayer membrane [51,52]. Camel MFGM proteins have been investigated in order to explain MFGM structure and how the proteins vary between the different species [47,48,49].

For example, Saadaoui et al. found 322 functional groups in the CM MFGM by SDS-PAGE separation and liquid chromatography–tandem mass spectrometry (LC–MS), with more than 50% of MFGM proteins being found to belong to the plasma membrane [49]. In 2015, Yang et al. compared the different mammalian MFG proteins using an isobaric tag for relative and absolute quantification (iTRAQ) proteomic approach. As a result, profiles of MFGM proteins from camels, horses, and humans were found to have a distinct composition compared to all the other studied species [47]. The study of Laadhar Karray et al. provides information on the chemical composition of camel MFGM (Figure 1B), its mechanical properties, and the effect of various temperatures on MFGM physical properties. The results showed that camel MFGM has a much lower protein content than cow MFGM, but it is a good source of PLs [29]. Hence, further studies are needed to investigate and comprehend the processing effect on the MFGM functionality, as well as the physical properties of MFGM in CM, which can be employed as a guide for food production.

Structurally, the MFGM consists of a PL monolayer derived from the endoplasmic reticulum and an outer bilayer, coming from the plasma membrane during budding, with varying concentrations of energized cytoplasm between the layers [53]. Microscopic examination showed that the MFGM in CM was thicker than that in cow milk [54,55], which contributed to camel milk’s improved stability in emulsions. However, the thickness of MFGM and relatively small size of fat globules made it difficult to separate the milk cream portion [56].

Finally, it has been suggested that MFGM proteins may be exploited to make products rich in MFGM fraction to boost the immune system, control cholesterol metabolism, and provide the body with beneficial polar lipids [57]. Moreover, the MFGM protects the triglycerides from lipolysis and auto-oxidation before the actual digestion [58]. As a result, MFGM protein is used in the dairy industry, particularly in infant formula supplements. In addition, it has numerous health-promoting properties, such as antiviral [59], anticancer [60], and anti-inflammatory effects [61]. Thus, future research will most likely focus on a deeper comprehension of MFGM proteins in CM as well as their possible biological roles.

### 3.2. CM Fat Globules

A significant component in milk that attracts attention is the MFG. It plays a prominent role in the nutrition and technology of dairy products. The MFG comprises a neutral fat core composed of 98.99% triacylglycerols (TAGs) [62]. Its biogenesis begins either inside the endoplasmic reticulum or in close proximity [63,64], further being transferred to the apical side of the cell upon release from the endoplasmic reticulum and it increases in size by MFGs fusion [65].

**Figure 3:**
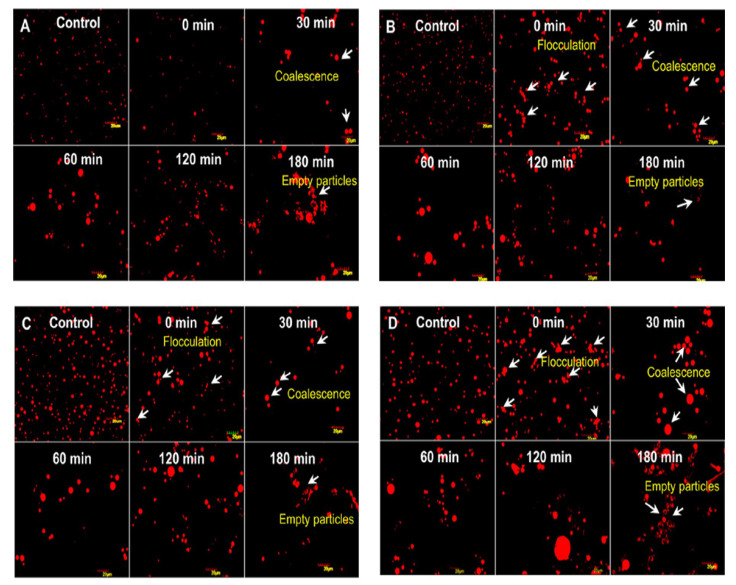
Comparison of different milk fat globules: camel (**A**), goat (**B**), cow (**C**), and buffalo (**D**) by confocal microscopy. Adapted with permission from Ref. [10]. Copyright 2014 Elsevier.

**Figure 4:**
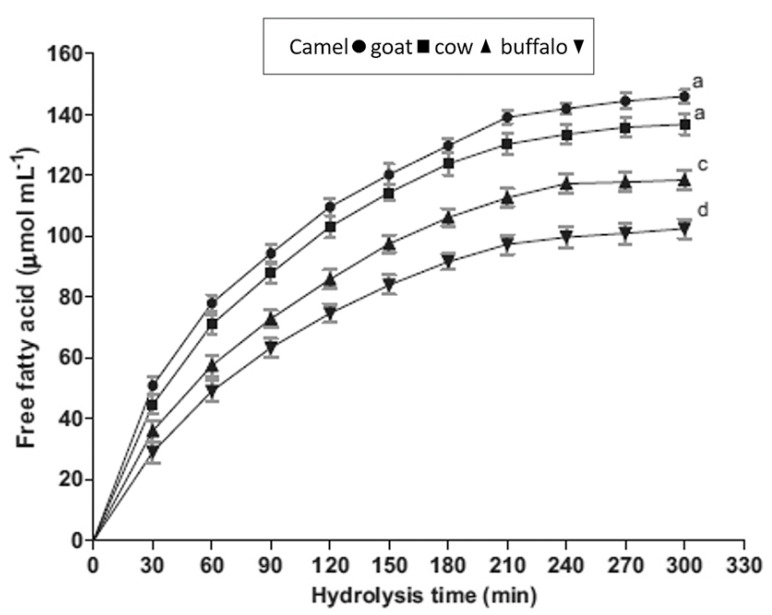
Comparison of the free fatty acid amount released during digestion from different kinds of milk. Reprinted with permission from Ref. [10]. Copyright 2014 Elsevier.

In the literature, much data about the average diameter of CM fat globules has been reported. According to Farah and Rüegg [55], it is 2.61 μm, whereas another study reported that roughly 55% of the population had a volume diameter of less than or equal to 2 μm [54]. El-Zeini et al. stated that most CM fat globules have a diameter of 2 to 4 μm, accounting for 61% of the population [66]. In 2020, Habtegebriel et al. used laser diffraction to study the colloidal size distribution of MFG [67], providing evidence that almost 50% of the population exhibited a size less than 2.68 μm. Summarizing the collected literature data, we find that the MFG diameter generally ranges from 0.1 to 10 mm, with an average value of about 4 mm, depending on animal breed, with the size of the fat droplet found to increase during lipid biosynthesis. Previous studies highlighted some profound differences in physical and chemical properties of the camel MFG compared to the other types of mammalian milk. These differences are mainly due to camel MFG being smaller in size in comparison to other milk-derived mammals [10,45,66]. For example, the average MFG size in *C.* *dromedarius* in India, measured using confocal laser scanning microscopy, was 1.1–2.1 mm, thus smaller than that of buffalo (3.9–7.7 mm), cow (1.6–4.9 mm), and goat (1.1–3.9 mm) milk [10]. A similar average MFG size was found in *C. bactrianus* milk fat (1.80 μm) [68]. It is important to prove that the size of the original MFG affects the physio-chemical properties and, in particular, the milk digestibility, which is inversely proportional to the size of the fat globules [69]. Indeed, in vitro study conducted by Meena et al. [10] showed that the CM fat has higher fat digestibility compared to the cow and buffalo milk due to the small size of MFG (Figure 3), taking into account that the fat digestion typically occurs in both the stomach and small intestine [58,70]. However, there was no significant difference (*p* > 0.05) among them in the release of FAs at the end of lipid digestion (Figure 4).

There is also a connection between the MFG size and the amount of FAs in the milk fat. CM, which has the smallest MFG size range among all the major dairy species [6,10,45,66], has a high level of UFAs and relatively little short-chain FAs [19]. These results suggest that UFAs is inversely connected to the size of the MFG in CM compared with other types of mammalian milk (Figure 2A) [45]. In general, variations in the phenotypes of small and large MFG reflected the differences in the FA content. It was also observed that the MFG size distribution for camels was similar to the MFG size distribution for other milk species [71]. The high state of dispersion of CM fats affects the lipolytic enzymes accessing the small fat globules, being easily digested by humans [71]. Other *C.* *dromedarius* MFG parameters, such as the fat globular volume, border, surface area, width, and length, were investigated and found to be 14 μm, 9.6 μm, 7.78 μm 1.65 μm, and 27%, respectively [66], whereas the properties of MFG in *C. bactrianus* milk have received less attention. The structure of MFGs and MFGM can be influenced by a variety of factors, such as the size of fat globules [72,73], which in turn may be affected by the lactation stages [74]. Jenness et al. reported that the size and numbers of fat globules in cow, buffalo, sheep, and goat milk are also affected during technological treatments [75].

From a technological point of view, the camel MFG behavior during drying has been recently studied by Zouari et al. [76]. The microstructure of drying the whole CM revealed less surface roughness compared to that of partially skimmed cow milk. Furthermore, the lower distribution size and the high crystallization temperature of CM fat led to the encapsulation of most CM fat globules with proteins near the surface of the powder.

## 4. CM Fat Composition

CM fat nutritionally serves as an energy source, acts as a solvent for the fat-soluble vitamins, and supplies EFAs. Thus, it is important to examine its structure and composition. CM fat accounts for 1.2–5.4% [77], with an average of 3.29%, of the total CM [39], and is mostly composed of TAGs, as well as cholesterols and PLs.

### 4.1. FAs Composition of CM Fat

The composition of FAs in milk fat is highly complex since they are derived from rumen microbial metabolism, body storage, and dietary FAs. FAs are typically categorized into three groups depending on their saturation level i.e., SFAs, MUFAs and PUFAs, which comprise *n*-6 PUFAs and *n-*3 PUFAs. In the next subsections, FA distribution in CM from 11 countries and 13 regions is described in connection to its physicochemical and nutritional value, with an emphasis on environmental and physiological variables affecting FAs. Several previous studies have been reported on the FA composition in CM, as summarized in Table 1 and Table 2.

### 4.2. Saturated Fatty Acids (SFAs)

SFAs are the most common FAs and are present in CM at a lower percentage when compared to cow milk (46–66% (Table 1) vs. 78.33% of total FAs [20]). The most predominant SFAs are C16:0, followed by C18:0 and C14:0. In terms of diet, C18 SFA has been found to have a neutral health impact, while C14 and C16 SFAs are considered harmful because they are linked to high serum low-density lipoprotein (LDL) cholesterol concentrations in human subjects [78]. High consumption of SFAs has negative health implications as it inhibits the metabolism of *n*-6 FAs and causes deficiency of EFAs [79]. Furthermore, a high intake of SFAs is associated with an increased risk of developing coronary heart diseases [80]. Among the detected (C10-C14) SFAs, there are significant concentrations of medium-chain FAs (MC-FAs). This is a beneficial aspect since MC-FAs are easier to digest and metabolize than LC-FAs [11]. Camels are ruminants that can produce (C4-C8) FAs by fermenting cellulose. The concentration of (C4-C8) SFAs in CM, however, is small compared to other ruminant species, such as sheep and goats. Possible explanations for this low concentration could be either due to the rapid metabolism by camel tissue before being excreted into milk [81] or the different nature of camel feeding. This imparts some interesting nutritional properties to CM, as the content of (C4-C8) SFAs has high degrees of similarity to human milk.

**Table 1:** Composition of saturated and monounsaturated FAs in camel milk from four different factors.

Locality/References		SE	L/Days	4:0	6:0	8:0	10:0	12:0	13:0	14:0	14:1	15:0	FAs 15:1	16:0	16:1	17:0	17:1	18:0	18:1 n-9	20:0	20:1	ΣSFAs	ΣMUFAs
China ** [36]		W	NS	12.20	0.01	0.26	0.21	0.91	0.08	12.00	0.55	1.09	0.36	23.60	0.42	0.56	0.49	12.80	15.15	0.08	0.08	64.10	25.70
China ** [82]		NS	NS	ND	0.10	0.10	0.10	0.89	ND	7.32	0.33	1.71	ND	18.80	3.51	0.89	ND	21.30	28.10	0.31	0.22	51.90	39.60
China ** [13]	Hovd	NS	NS	0.02	0.20	0.09	0.01	0.81	0.01	10.18	0.27	1.94	ND	23.99	5.58	1.16	0.66	16.18	22.42	1.72	ND	57.53	34.15
	Sharga	NS	NS	0.01	0.10	0.13	0.11	1.09	0.03	12.68	0.42	1.71	ND	30.08	5.37	1.21	0.56	16.49	15.79	0.89	ND	65.24	26.14
	Tseel	NS	NS	0.01	0.12	0.10	0.05	0.83	ND	12.24	0.42	2.14	ND	27.40	5.72	1.46	0.76	14.99	17.87	1.09	ND	61.12	29.16
	Bulgan	NS	NS	0.04	0.30	0.36	0.16	0.98	0.03	13.15	0.62	2.23	ND	30.48	7.53	1.29	0.79	11.13	18.13	0.39	ND	60.90	30.13
	Tsogtovoo	NS	NS	0.04	0.22	0.29	0.18	1.55	0.06	13.66	0.58	1.94	ND	30.72	7.40	1.22	0.63	12.27	17.06	0.56	ND	62.75	29.29
China ** [83]		NS	NS	ND	ND	ND	ND	0.78	ND	11.49	ND	ND	ND	30.12	ND	ND	ND	15.15	26.05	ND	ND	NS	NS
China ** [84]		NS	NS	ND	ND	ND	ND	0.96	0.05	12.50	0.58	1.23	ND	31.92	7.32	ND	ND	16.10	21.87	ND	ND	NS	NS
China ** [12]		W	NS	ND	ND	ND	ND	0.83	ND	11.54	1.56	1.57	ND	31.51	5.17	ND	ND	18.67	23.59	0.31	0.08	64.42	30.40
Turkey [18]		NS	NS	0.02	0.18	0.18	0.32	0.02	ND	10.84	ND	ND	ND	24.90	11.86	ND	ND	15.38	30.74	0.66	ND	NS	NS
Germany [35]		S	NS	ND	ND	ND	ND	0.43	0.20	10.50	1.17	1.71	0.07	27.60	10.70	0.82	0.64	12.50	17.2	0.42	0.11	54.50	32.20
China ** [85]		NS	NS	ND	ND	ND	ND	0.88	ND	13.04	1.49	1.06	1.64	34.13	8.85	0.92	0.82	14.58	18.96	ND	ND	64.61	31.76
China ** [20]		NS	NS	ND	ND	ND	ND	1.05	ND	11.84	0.72	ND	ND	27.07	9.74	ND	ND	11.85	29.25	0.63	0.14	53.66	41.00
Kazakhstan ** [11]		NS	NS	0.54	0.46	0.53	0.46	1.24	0.17	15.43	0.80	1.41	ND	32.05	7.01	0.65	0.33	14.75	18.78	0.01	ND	NS	NS
Kazakhstan * [11]		NS	NS	0.34	0.29	0.27	0.27	0.80	0.03	10.10	0.57	1.24	ND	29.74	6.60	0.76	0.38	17.82	24.66	0.05	0.01	NS	NS
UAE * [19]		NS	NS	ND	ND	ND	0.54	1.21	0.09	15.84	2.16	1.46	ND	35.97	10.07	0.80	0.49	11.55	16.34	0.46	ND	46.41	49.33
Saudi Arabia * [86]		W	90	0.11	0.90	0.22	0.23	1.54	ND	15.89	ND	1.39	ND	34.65	11.87	0.58	0.64	8.88	15.44	ND	0.23	66.40	30.30
Jordan * [87]	AlKhalidyah	S	NS	0.08	1.90	0.14	0.11	0.70	0.07	8.84	0.58	1.47	0.54	29.86	7.18	0.74	0.81	13.76	26.25	0.045	2.01	59.81	NS
	Al Hazeem			0.10	2.90	1.40	1.09	3.42	2.50	11.23	1.96	0.40	0.30	18.16	5.93	0.64	1.26	9.18	24.74	0.045	2.64	53.62	NS
	Al Umari			0.07	0.41	0.13	0.22	0.93	0.06	7.79	0.85	1.55	0.08	24.66	9.19	0.68	0.88	12.70	32.88	0.25	3.13	50.15	NS
	Al Safawi			0.05	3.30	1.30	1.04	2.58	1.00	13.32	1.70	2.14	0.15	25.00	11.54	0.64	1.18	8.70	22.52	0.14	1.26	61.16	NS
	Al Hamra			0.03	0.50	0.23	0.50	1.01	0.04	9.17	0.73	0.96	0.02	32.48	9.47	0.53	0.75	9.13	32.48	0.08	0.23	55.25	NS
	Al Qatrana			0.10	3.80	1.58	1.25	0.90	0.09	14.28	2.14	1.01	0.07	32.43	16.02	0.04	0.53	6.96	16.64	0.14	0.17	64.12	NS
	Wadi Araba			0.21	3.60	0.43	1.80	1.35	0.18	8.86	0.51	2.47	1.17	24.30	7.01	1.53	3.60	15.20	26.37	0.15	0.29	60.58	NS
	Al Jweideh			0.22	1.90	2.44	1.88	1.58	0.20	7.64	1.14	1.33	0.61	23.80	10.47	0.60	1.32	11.92	20.36	3.95	1.98	58.66	NS
Maghrebi * [88]		NS	140	ND	0.10	0.10	0.10	0.70	0.10	9.90	0.70	1.50	ND	28.70	8.10	0.10	ND	13.40	25.30	0.50	0.20	56.00	39.00
			245	ND	0.10	0.20	0.20	0.90	0.10	14.60	1.20	1.40	ND	37.20	10.90	0.05	ND	9.60	15.20	0.40	0.20	66.00	31.00
			329	ND	0.10	0.20	0.20	1.10	0.10	15.30	2.00	1.50	ND	31.20	13.40	0.06	ND	7.70	17.90	0.40	0.20	59.00	37.00
Egypt * [12]		W	NS	ND	ND	ND	ND	0.83	ND	10.55	2.33	1.64	0.75	27.90	7.56	0.79	0.75	12.99	29.83	0.28	ND	54.98	41.22
Egypt * [89]		NS	NS	0.83	0.37	0.28	0.37	0.66	ND	10.98	1.49	ND	ND	29.05	10.13	ND	ND	12.38	24.45	0.70	ND	NS	NS
Sudan * [90]		S	NS	ND	0.20	ND	0.12	0.41	0.13	8.43	0.58	1.05	ND	30.74	7.81	0.67	0.40	21.11	24.21	0.48	0.13	63.83	34.38
Tunisia * [91]		W	100–180	ND	ND	0.50	0.30	1.66	0.06	14.91	0.90	2.03	0.28	28.50	6.34	0.91	0.57	10.52	18.39	0.24	0.66	62.84	33.35
Tunisia * [92]		MJ	28–56	0.06	0.17	0.14	0.18	0.86	ND	11.65	1.49	ND	ND	31.26	10.03	ND	ND	10.30	17.38	0.06	0.06	60.31	32.88
			77–175	0.04	0.11	0.09	0.12	0.75	ND	9.05	1.34	ND	ND	27.63	7.54	ND	ND	12.45	23.78	0.09	0.09	55.49	37.17
			196–366	0.05	0.13	0.15	0.20	1.02	ND	11.50	1.52	ND	ND	28.22	8.50	ND	ND	11.46	19.05	0.03	0.08	58.50	33.25

Lactation (L), season (SE), United Arab Emirates (UAE), winter (W), summer (S), March 2015 to January 2016 (MJ), saturated fatty acids (SFAs), monounsaturated fatty acids (MUFAs), polyunsaturated fatty acids (PUFAs). Locality * present only in *C. dromedarius*. Locality ** present only in *C. bactrianus*. ND: not detected; NS: not specified.

### 4.3. Monounsaturated Fatty Acids (MUFAs)

MUFAs are the second kind of FAs found in CM fat, mostly represented by oleic acid (18:1 *n*-9), which accounts for 5.15–32.88% of overall FAs composition (Table 1), followed by palmitoleic acid (16:1). The MUFA content of CM fat is slightly higher than that of other mammalian species milk fats [20]. The higher level of MUFAs in CM can be explained either by the slower hindgut fermentation or by the higher FAs desaturase activity, which is responsible for the biosynthesis of MUFAs and PUFAs in CM. These latter data may provide a significant nutritional index, because MUFAs have beneficial effects on arterial diseases, lowering plasma LDL cholesterol and total cholesterol as well as the fibrinolytic activity of circulating plasma by modifying vascular endothelial physiology [93].

### 4.4. Polyunsaturated Fatty Acids (PUFAs)

PUFAs account for 2.7–8.46% of the total FAs in CM (Table 2), which is higher than cow milk (1.89%) [20] but still less than human milk (10–20%) [94]. Owing to bacterial biohydrogenation in the rumen, ruminants have generally low levels of PUFAs. It should be noted that PUFAs play a pivotal role in the growth of the neonatal brain, as well as the retina and cognitive functions. Among PUFAs, LA (C18:2 n-6) and ALA are the major *n*-6 PUFA and *n*-3 PUFA, respectively. LA is within the range of 0.17–3.31%, while ALA is within the range of 0.05–2.16% of total FAs. The percentage of LA in CM is 4–16-fold lower than in human milk [94], albeit the percentage of LA in CM is still higher than that in cow milk (i.e., 1.12% [20]).

ALA is the major omega-3 FA in milk: the proportion of ALA was found to be one fold higher in CM than in human milk and from 10 to 13 fold higher than in cow milk [20,36], which has been linked to its antiarrhythmic effects, positive influence on neurological activity (by reducing central nervous system injury), and protective effects against coronary heart disease [95]. CM fat also contains conjugated linoleic acid (CLA), with several various isomers, which is produced during the biohydrogenation process of ruminants [15]. Rumenic acid (RA; C18:2, cis-9, trans-11) and t10 c12 are the two major CLA isomers found in CM, accounting for 0.80 ± 0.15 and 0.06 ± 0.02 of total FAs, respectively [81]. Compared with human milk, CM fats were higher in CLA contents [35,36]. It has been reported that CLA showed a cytotoxic effect on cancer cells [96], indicating that it is beneficial to human health.

EPA, docosahexaenoic acid (DHA), and AA are among the PUFAs that were observed in minute quantities (Table 2). The percentage of EPA, DHA, and AA in CM were reported to be lower than that in human milk (1%) [94]. In contrast, one study revealed that CM had a higher amount of EPA and AA (0.14 and 1.35 g/100 g, respectively) than human milk (0.03 and 0.67 g/100 g, respectively) [36]. However, these results must be further confirmed by conducting additional studies on both camel and human milk. In addition, the determination of LC–PUFA metabolites from both animal and human milk samples is very difficult because it needs a special chromatography column, and the amounts of these FAs are near the limit of quantitation (LOQ).

**Table 2:** Composition of polyunsaturated FAs in camel milk from four different factors.

Locality/References		SE	L/days	18:2 *n*-6tt	CLA	18:2 *n*-6	18:3 *n*-6	FAs 20:3 *n*-6	20:4 *n*-6 (AA)	18:3 *n*-3	20:5 *n*-3 (EPA)	22:5 *n*-3	22:6 *n*-3 (DHA)	ΣPUFAs
China ** [36]		W	NS	0.29	0.59	3.19	0.01	0.09	1.35	2.12	0.14	0.34	0.01	8.10
China ** [82]		NS	NS	1.85	4.5	2.66	0.53	0.53	ND	1.81	ND	ND	ND	8.46
China ** [13]	Hovd	NS	NS	ND	ND	2.54	ND	ND	ND	1.13	ND	ND	ND	4.42
	Sharga	NS	NS	ND	ND	1.92	ND	ND	ND	1.21	ND	ND	ND	3.76
	Tseel	NS	NS	ND	ND	2.21	ND	ND	ND	1.03	ND	ND	ND	3.97
	Bulgan	NS	NS	ND	ND	1.73	ND	ND	ND	0.61	ND	ND	ND	2.94
	Tsogtovoo	NS	NS	ND	ND	1.68	ND	ND	ND	0.46	ND	ND	ND	2.70
China ** [83]		NS	NS	ND	ND	2.04	ND	ND	ND	2.04	ND	ND	ND	NS
China ** [84]		NS	NS	ND	ND	1.51	ND	ND	ND	1.51	ND	ND	ND	NS
China ** [12]		W	NS	0.26	ND	4.09	0.66	ND	ND	0.05	ND	ND	ND	5.18
Turkey [18]		NS	NS	ND	ND	2.12	ND	ND	ND	1.74	ND	ND	ND	NS
Germany [35]		S	NS	0.47	0.31	3.07	ND	0.12	0.21	0.17	0.01	ND	0.02	3.91
China ** [85]		NS	NS	ND	ND	2.28	ND	ND	ND	1.31	ND	ND	ND	3.69
China ** [20]		NS	NS	0.18	ND	3.31	0.17	0.36	ND	1.37	ND	ND	ND	5.21
Kazakhstan ** [11]		NS	NS	ND	ND	1.19	ND	ND	ND	0.60	ND	ND	ND	NS
Kazakhstan * [11]		NS	NS	ND	ND	1.61	ND	ND	ND	0.51	ND	ND	ND	NS
UAE * [19]		NS	NS	0.49	ND	1.73	0.21	ND	ND	0.26	ND	ND	ND	4.26
Saudi Arabia * [86]		W	90	ND	0.23	2.14	0.28	ND	ND	0.51	0.05	ND	0.19	3.40
Jordan * [87]	AlKhalidyah	S	NS	1.12	ND	2.00	0.01	ND	ND	1.63	ND	ND	ND	NS
	Al Hazeem			1.25	ND	2.60	2.17	ND	ND	6.15	ND	ND	ND	NS
	Al Umari			1.56	ND	3.10	0.10	ND	ND	1.19	ND	ND	ND	NS
	Al Safawi			0.06	ND	1.20	0.04	ND	ND	0.33	ND	ND	ND	NS
	Al Hamra			0.46	ND	0.23	0.005	ND	ND	0.58	ND	ND	ND	NS
	Al Qatrana			0.15	ND	0.17	0.06	ND	ND	0.09	ND	ND	ND	NS
	Wadi Araba			0.20	ND	0.29	0.11	ND	ND	0.16	ND	ND	ND	NS
	Al Jweideh			1.25	ND	1.98	1.44	ND	ND	2.78	ND	ND	ND	NS
Maghrebi * [88]		NS	140	2.70	0.9	2.70	ND	0.1	0.3	0.50	ND	ND	0.2	5.10
			245	2.00	0.4	2.00	ND	0.1	0.2	0.50	0.1	ND	0.1	3.80
			329	2.40	0.7	2.40	ND	0.1	0.2	0.50	0.1	ND	0.2	4.50
Egypt * [12]		W	NS	0.46	ND	3.21	ND	ND	ND	ND	ND	ND	ND	3.67
Egypt * [89]		NS	NS	ND	ND	3.11	ND	ND	ND	1.39	ND	ND	ND	NS
Sudan * [90]		S	NS	0.12	ND	1.40	ND	ND	ND	0.13	ND	ND	ND	1.78
Tunisia * [91]		W	100–180	0.25	2.05	2.05	ND	ND	0.04	1.24	ND	ND	ND	3.58
Tunisia * [92]		MJ	28–56	ND	0.21	2.07	ND	0.18	0.04	ND	ND	ND	ND	5.12
			77–175	ND	0.29	2.12	ND	0.22	0.09	ND	ND	ND	ND	5.44
			196–366	ND	0.49	2.20	ND	0.18	0.1	ND	ND	ND	ND	5.97

Lactation (L), season (SE), United Arab Emirates (UAE), winter (W), summer (S), March 2015 to January 2016 (MJ), saturated fatty acids (SFAs), monounsaturated fatty acids (MUFAs), polyunsaturated fatty acids (PUFAs). Locality * present only in *C. dromedarius*. Locality ** present only in *C. bactrianus*. ND: not detected; NS: not specified.

It is worth noting that one study reported that CM fats had higher levels of EPA and AA than cow milk fats [36], while another study found no EPA or AA in CM fats [20]. This discrepancy could have been due to different authors using different analytical techniques and measurement units. Some studies reported that DHA of both CM fats existed in small amounts lower than 0.2% [35,36,86,88,97,98,99,100]; however, other studies have found no evidence of presence of DHA. Nevertheless, CM can still be used in infant nutrition, but addition of AA and DHA are needed, as their metabolism is limited [79,101]. Overall, most of the literature findings for the FA composition in CM (15–16 studies) showed no evidence for presence of EPA, AA, and DHA. In contrast, only 4–5 studies reported their presence in small quantities (Table 2). Of note, these frequencies are very similar to the presence of SC-SFA.

There are some differences between CM and human milk in terms of FA content. Despite this difference, Egyptian nomads often use CM to feed their children [5]. Furthermore, CM can be supplemented either with vegetable oil or through the enzymatic/chemical modification of fat to become more comparable to human milk fat, and this method is currently the most widely used by infant formula manufacturers. Generally speaking, CM fats were, to some extent, different from other ruminant and human milk fats with a lower number of FAs and higher levels of 16:1, 18:1, CLA, and ALA acids. Furthermore, CM showed a better unsaturated/saturated acid ratio than milk from other mammals [11], posing it as a potential superfood, although currently being unrecognized as such.

### 4.5. Environmental and Physiological Factors Affecting FA Composition in CM

**Figure 5:**
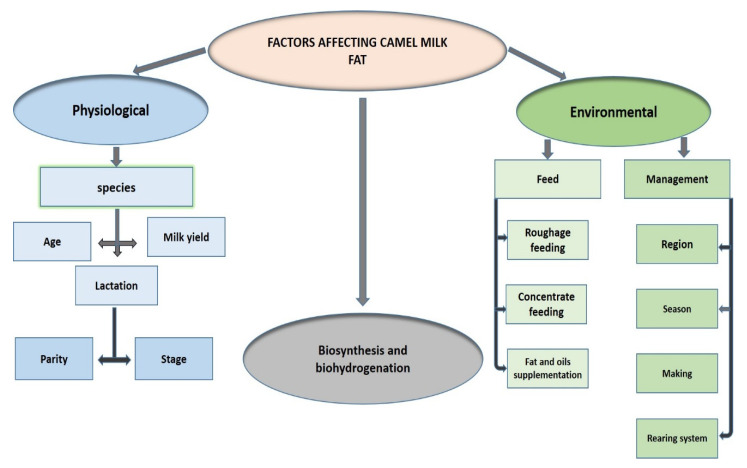
Factors affecting camel milk fat composition.

The CM FA composition has distinctive properties and is influenced by many environmental and physiological factors, as summarized in Table 1 and Table 2 and Figure 5. Four variables related to the FAs composition have been the most investigated: region, species, season, and lactation stage. FA content of CM fat was found to differ depending on the country where the camels live [102], as well as on the geographic region within the countries [11,13,87].

SFAs and PUFAs constitute 60.60% and 4.80% of overall FAs, respectively, in *C. bactrianus* milk fat, which is higher than SFAs (56.03%) and PUFAs (4.20%) in *C. dromedarius* milk fat. While *C. dromedarius* milk fat contains more MUFAs (36.70%) than *C. bactrianus* CM fat (31.40%), as reported by Zhao et al. [103]. The FA composition was studied without taking into account the variations due to seasonal conditions in the majority of the literature data [11,12,14,15,18,20,54,68,88,97,102,104], suggesting that this aspect should be considered in the future. Yet, short-chain FAs (C8:0 and C10:0) were found to be more abundant in the spring, while LC-FAs (C17:0 and C17:1) were found to be more abundant in the autumn [11]. The stage of lactation is one of the most significant factors influencing FA composition, which is divided into colostrum (1–5 days), intermediate milk (6–15 days), and mature milk (>15 days). The average lipid content of the CM colostrum (30.1–19.5 g/L) is lower than that of mature milk (32.8–14 g/L) [97]. The study also indicated that SFA levels tend to increase during lactation, concurrent with a decrease in UFA levels. To the best of our knowledge, the information about the impact of both the season and the stage of lactation on the FA composition of *C. bactrianus* milk has not been documented yet.

### 4.6. Physical Characteristics of the CM Fat

Milk fat has a wide range of physical characteristics since it includes over 400 different FAs. Nevertheless, it is mostly composed of 16 major FAs that are responsible for its physical properties, i.e., melting and solidification temperatures, solid-phase content, firmness/hardness, and spreadability of the resulting butter [14,105]. Table 3 shows that the acid and saponification values of *C. dromedarius* milk fat are significantly higher than those of *C. bactrianus* milk fat. However, the CM fat value is substantially lower than the cow milk fat value, likely attributed to the presence of more LC-FAs in CM fat. *C. bactrianus* milk fat shows a higher refractive index and iodine value than *C. dromedarius* milk fat, with both values in camels higher than cow milk fat (Table 3).

**Figure 6:**
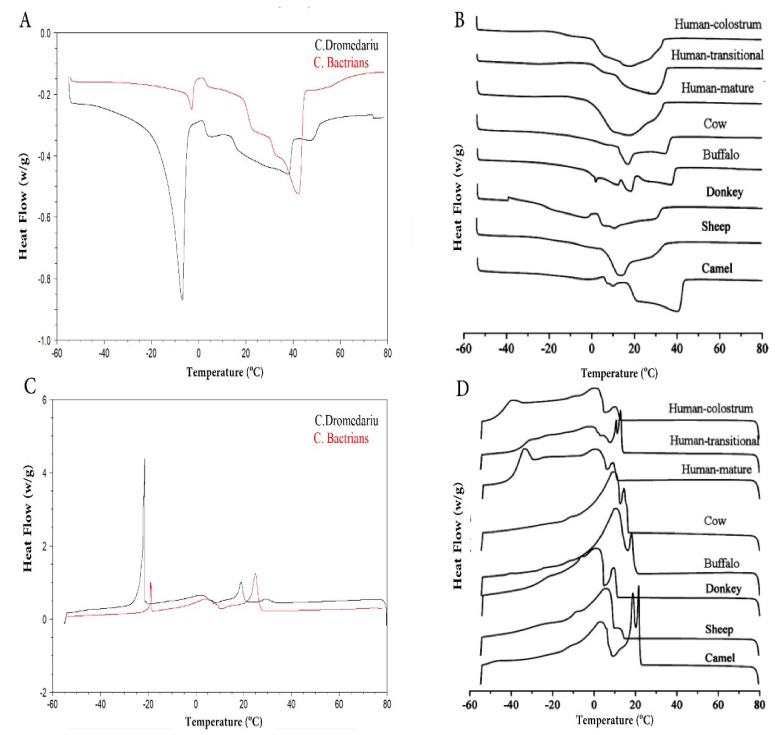
DSC curves of crystallization (**A**,**B**) and melting (**C**,**D**) for camel milk and other mammalian milk. Adapted with permission from Ref. [12] (Copyright 2020 Elsevier) (**A**,**C**) and Ref. [20] (Copyright 2013 ACS Publications) (**B**,**D**).

This indicates that CM fat contains higher UFAs levels compared to cow milk fat. Figure 6 shows the differences in the differential scanning calorimetry (DSC) profiles among both camels and other mammalian milk. The detected differences can be attributed to the FAs/TAG composition. Thus, the melting point of the fats decreases with decreasing chain length and increasing the degree of unsaturation of the FAs [108]. *C. bactrianus* milk fat showed a higher melting point than *C. dromedarius* milk fat, both higher in camels than in cow milk fat. This may be due to the high amount of large TAGs (Carbon number, CN48 -CN 52) [12,14] concurrent with the low proportion of short-chain FAs and UFAs in CM fat [5]. A spectroscopic technique, i.e., Fourier transform infrared spectroscopy (FT-IR), was able to distinguish between both CM fat samples [12]. The use of different examination techniques (FT-IR, DSC) for CM fat can be advantageous in comparing it with other types of milk fat.

## 5. Composition and Distributions of TAGs in the CM

### 5.1. TAGs Composition

The determination of CM fat physicochemical and nutritional properties provides insight into the structure of TAGs, which is defined by the types, quantities, and distribution of FAs. Differences in diet, season, lactation stage, and animal species can lead to changes in the amount and composition of milk TAGs. Only one study reported on the composition of milk TAGs for both types of camel milk [12]. Previous studies of TAGs in milk fat have shown that there are a large number of species-related TAGs, as well as the associated positional isomers, which makes the determination of TAG composition rather challenging [12,15]. For example, there were few variations among the species; for example, *C. bactrianus* milk fat had more TAGs than *C. dromedarius* milk fat. The species influence has been attributed to the empirically observed positive association between TAG saturation and relative strength of [TAG^+^H]^+^ in a variety of molecular systems, most notably in the relative splint magnitude of a sequence of linolyte and arachidonate TAGs [109]. In addition, TAGs composition in CM significantly differs from that of other ruminants [110]. Several studies [12,14,20,85,91] showed that major TAGs in CM fat are CN46, CN48, CN50, and CN52 (Table 4) and that CM fat is free of SC-SFAs and characterized by low MC-SFAs levels, which may be attributed to the lower levels of caprylic and capric acids in the lipids. As a result, TAGs in this milk fat are often made up of LC-FAs with high equivalent carbon atom numbers. In addition, Ref.[14] found that TAGs composition had a similar trend in buffalo, cow, and sheep milk, which were found to be richer in CN32 and CN40 fats than in CM.

Odd-chain FAs are widely present in CM fat and constitute about 13.5% of TAGs [91], allowing for several nutritional and medical applications [111]. The majority of TAGs in the mammary glands of camels were found to be made up of both SFAs and UFAs [91], likely due to the preservation of fat fluidity at physiological temperatures. According to a previous study by Haddad et al. [91], CM contains a large amount of saturated and unsaturated LC-TAG molecular species. From an industry point of view, these saturated LC-TAG molecular species improve the crystallization of butter products [112], and it would be very important to examine whether its inclusion in butter manufacture would improve quality without affecting other sensory characters. In contrast, the high degree of unsaturated LC-TAG molecular species is negatively associated with firmness in bovine milk butter [113].

### 5.2. FA Distribution on TAGs

To date, relatively few studies have reported TAG distribution and composition in CM compared to extensive reports focused on cow and human milk. The acyltransferase specificity and activity are responsible for the non-random FA distribution. Thus, by comparing experimental to theoretical TAG distributions, researchers have shown the non-random distribution of FAs in CM [91]. FA distribution of CM TGA follows a certain pattern with the highest concentration of SFAs detected at the *sn*-2 position, whereas UFAs are primarily at *sn*-1 and *sn*-3 positions. The majority of palmitic acid is present at the *sn*-1,3 locations [12,15]. Thus, there are relatively low contents of palmitic acid at the *sn*-2 positions, contrary to most natural fats. As a result, the distribution of the three main FAs in natural fats significantly differs from that in human milk and standard vegetable oils [94], whereas the presence of palmitic acid at the TAG *sn*-2 location has been linked to the enhanced fat and calcium absorption, intestinal comfort, and the growth of intestinal microorganisms [114,115]. Other properties of CM TAG distributions are primarily responsible for the rheological qualities of milk fat products.

## 6. Composition and Nutritional Properties of CM Cholesterol

The most abundant sterol in CM fat is cholesterol, whose level is determined by the overall fat content in milk [116]. Cholesterol is an important lipid ingredient for human survival that is biosynthesized in the body or consumed from an external source. Its content in milk has attracted great attention from medical research, and it is often linked to coronary heart diseases [23]. However, it has been reported that more research is required to fully understand the impact of cholesterol on health, especially given that its bioavailability is rather limited to exogenous sources [117]. The average cholesterol level of CM (5.64–3.18 mg/100 g) was found to be lower than that of cow milk (8.51–9.07 mg/100 g). Breed, species, cholesterol/fat ratio, age, diet, milking time, and lactation stage are all factors that influence the cholesterol level in CM [11,14,23,24,86,118,119]. It has been reported that the cholesterol level in CM changes with the lactation stages. Gorban and Izzeldin [24] found that the total cholesterol in camel colostrum is higher (27.6 mg/100 mL) than in mature milk (31.3 mg/100 mL), while Kamal and Salama [119] found that the cholesterol level was higher in colostrum on day 1 post-partum (44.5 mg/100 mL) than day 30 post-partum (18.9 mg/100 mL), as expected.

To date, CM cholesterol has been a subject of debate because it appears to be completely different from that of the cow milk, especially in terms of the total cholesterol level. The cholesterol level of CM fat was found to be lower than that of cow milk fat [14,23], also characterized by richness in SFAs compared to CM, another predisposing factor that can increase overall cholesterol levels in the human blood [120].

On the other hand, a greater proportion of small fat globules is associated with a higher concentration of cholesterol in milk, due to the greater surface area of MFGM per fat unit [72]. Following such a hypothesis, [11,24] demonstrated that the CM showed a higher cholesterol level (31.3 to 37.1 mg/100 g) compared to cow milk fat, in addition to its doubled fat amount compared to cow milk [11]. Nevertheless, Faye et al. reported higher fat levels in cow milk than in camel milk, while the cholesterol levels of both types were similar [23]. Considering the cholesterol/fat ratio instead of the cholesterol/milk ratio, researchers found that the cholesterol content was higher in cow milk than in CM [14]. Thus, much discrepancy in the data has been reported and as has already been proven, that cholesterol content in CM is a controversial topic. Indeed, the authors of different works have used various analytical techniques and measurement units, making a proper cholesterol concentration comparison among the different types of mammal milk very difficult. In the future, it would be important to compare the cholesterol concentrations in various mammals using the same analytical method, as well as through taking into account the variability of fat content in milk.

From a nutritional point of view, earlier studies have shown that the CM (fresh or fermented milk) exhibits a hypocholesterolemic effect on animals’ lipid profiles [25,26,27,28]. On the basis of the evidence of previous research on CM usage in the control of cholesterol levels, it can be inferred that CM plays a significant role in lowering cholesterol levels and that human intake of CM is beneficial to human health in the long term. However, the mechanism of the CM hypocholesterolemic effect is unknown to date, and different hypotheses have been proposed [121,122,123], suggesting the need for more and accurate work to clarify this issue.

## 7. Composition and Nutritional Properties of CM Phospholipids

PLs are one of the essential lipid components that can be used as functional ingredients by industries involved in either agro-food or clinical nutrition. The PL content in CM has not received much attention in comparison with human and cow milk. The major PLs present in camel MFGM were found to be phosphatidylcholine (PC), phosphatidylethanolamine (PE), sphingomyelin (SM), phosphatidylinositol (PI), and phosphatidylserine (PS), similar to those reported in cow MFGM [6,97,110,124]. During the first weeks of lactation, PL content in CM was slightly higher in mature milk (1.21%) than in colostrum (0.67%) [97]. To the best of our knowledge, various other factors that affect PL composition, such as genetics and nutrition aspects, have not been studied to date. SM plays a positive role in the mental, motor, and behavioral development of executive function during infancy [125]. Moreover, camel and human milk are richer in SM (117.5 and 78.3 µg/mL, respectively) and plasmalogens (24 and 27.3 µg/mL, respectively) [6], which makes CM the second source of plasmalogens after human milk. Dietary plasmalogens are absorbed by the intestine [126], and they have beneficial effects on the health of infants. CM serves as a promising dietary source of PI, PS, SM, PE, and plasmalogens relative to other milk species for both infants and adults.

Table 5 summaries the PLs contents and the different analytical methods used for the CM PL evaluation. Comparative studies have shown that thin-layer chromatography (TLC), high-performance liquid chromatography (HPLC), and ^31^P nuclear magnetic resonance (^31^P NMR) methods demonstrated comparable results for evaluating PLs in dietary matrices [127]. Morrison et al. isolated PLs in the CM for the first time, as well as from other different mammalian species, by TLC [124]. They found that the PL distribution and total content in the CM were remarkably regular and almost constant, and similar behavior was detected for all the studied species. Nevertheless, it is a good source of PE and plasmalogen. Conversely, FAs in CM PLs are not completely as characteristic as ruminant herbivores.

According to the literature, the ^31^P NMR spectroscopy method succeeded in identifying and measuring 12 PLs, including some lysophospholipids of CM, human milk, cow milk, and mare milk, with ease of sample preparation, high precision, and strong sensitivity [6]. It was found that the PL content in CM (0.503 mM) was higher than in human milk (0.324 mM), cow milk (0.265 mM), and mare milk (0.101 mM). Thus, taking into account that the dietary PLs are usually consumed in limited quantities, around 2–5 g per day [70], the research found that the CM is a potential new source of PLs.

Zou et al. [20] compared the PL profiles of milk from camel and other animal species using high-performance liquid chromatography (RP-HPLC). They discovered that the SM and PS contents of cow, buffalo, donkey, sheep, and camel milk fats were substantially lower than that of human milk, albeit PI content (6.05%) of CM fat was comparable to that of human milk, rationalizing the CM hypocholesterolemic effect [129]. With regards to FA composition in PLs, SFAs, LC-SFAs, and C16:0 levels in CM, PLs were significantly lower than in human milk, whereas no significant difference in the content of C18:0 was observed in camel and other milk fats. Apart from cow milk fat, the contents of MUFAs (33%) and LC-PUFAs (22.4%) in PLs of CM fats were significantly higher than in human milk (MUFAs 15.6% and LC-PUFAs 17.6%). Furthermore, LC-FAs, i.e., DHA and AA, were not found in PLs of CM fats. In a recent study, [110] provided detailed information on PL molecular species in CM. Six classes of PLs were detected using ultra-performance liquid chromatography–electrospray ionization–quadrupole time of flight–mass spectrometry (UPLC–ESI–QTOF–MS), including PC (C16:0-C18:1), PE (C18:0-C18:2), PI (C18:0-C18:1), PS (C14:0-C18:3+Na), SM (d18:1/16:0), and LPC (C16:0). This method was rapid, sensitive, and precise for the identification of milk PLs, and it could be used to investigate dairy products, as well as to investigate innovative uses of milk PLs. Ali et al. [110] also reported that the FA compositions of PLs in CM had a significantly lower content of SFAs than in other milk powder fats, and were more enriched in PUFAs. Thus, the fluidity/rigidity of MFGM is affected by the length and the degree of saturation of FAs in milk PLs through a functional role in the separation of the system membrane phase [130].

Briefly, studies presented here indicate that the content of PLs in CM was higher than in all other studied animal species. Nevertheless, the major PLs present in the CM MFGM are PC, PE, SM, PI, and PS, similarly to those in all studied species. On the basis of composition, CM exerts a better emulsifier action than human and cow milk, since it contains almost 1% of total lipids as PLs [6]. Thus, CM can replace human milk as it provides similar amounts of plasmalogens and SM, being essential during early life, to newborns [131]. Newborns are more susceptible to oxidative stress because they have lower erythrocyte levels of plasmalogens than older children [132].

## 8. CM Butter and Its Production Challenges

### 8.1. CM Butter

CM butter is one of the dairy products made from CM. In various regions around the world, such as Algerian Sahara [133], northern Kenya [134], and Sinai Peninsula [102], it is produced by a traditional churning process as in the case of other animal butter. Ghee (filtered butter) is produced from CM, a popular Indian commodity, though the end product has a lower yield than buffalo or cow milk [135]. Moreover, the possibility of making butter from CM was also reported by [102,136]. To produce 80% of butter yield, vigorous hot shaking (22–23 °C) was needed [102]. In 2021, Mtibaa et al. studied the influence of ripening and churning circumstances on the turn ability of CM cream and the physical characteristics of CM butter [137]. It was found that CM butter could only be produced when churned at 21 °C, independent of the ripening temperature. In arid areas, this commodity is essential for nutrition, and there is an increasing demand for it all over the world for its health benefits.

### 8.2. Physicochemical Properties of the CM Butter

Butter flavor and aroma, as well as its rheological properties, have a large impact on consumer acceptance. Its properties are determined by the types of FAs found in butter. SC-FAs play a significant part in butter flavor. The lower content of butyric acid in CM butter resulted in a less intense flavor relative to cow milk butter [138]. Another lacking chemical in camel butter is B-carotene, and thus the obtained butter is whiter in color compared to the cow milk-derived butter [102,104]. CM butter, on the other hand, contained a significant amount of MUFAs (C16:1 and C18:1) (32.2% of total FAs), which is nutritionally advantageous [138]. The butter characteristics are determined by the kinds of FAs present. Hard butter is made from fats with a high content of high melting point FAs, whereas soft butter is made from fats with a low quantity of low melting point FAs [139]. The rheological qualities of butter are largely determined by the TAG structure, mainly by the distribution of FAs at the *sn*-1, *sn*-2, and *sn*-3 positions, as well as by the ripening temperature [137]. The content of total solids and fat of *C. dromedarius* milk butter generally ranges from 64 to 65% and 49 to 58%, respectively [133,138]. Due to the high content of SFAs in CM cream, the churning temperature (15–35 °C) was found to be higher than that of cow milk cream (10–15 °C) [67]. As a result, the ripening and churning conditions had a significant impact on the melting characteristics and rheological behavior of CM butter [137]. The acid degree value, melting point, and refractive index for *C. dromedarius* were at 6.7, 43.2 °C, and 1.4, respectively [138]. Such values confirm that *C. dromedarius* milk butter is less prone to rancidity because of the lower acid degree value. The high level of LC-FAs (C14-C18) and low percentage of SC-FAs (C4-C6) in *C. dromedarius* milk butter may have contributed to its high melting point, and high refractive index value.

### 8.3. The Challenges of Industry Development of CM Butter

The selected technology for the manufacturing of CM butter is a major challenge. Indeed, traditional churning presents some criticisms because it shows a slight tendency to cream up, due to the lack of the agglutinin protein in the CM [62]. The euglobulin protein is the key factor responsible for speeding the cream layer development in cow milk. Consequently, CM displays a very slow creaming rate at all temperatures with respect to cow milk [55]. Camel MFGs and proteins are closely related, which is an inherent property of the CM fat [140]. Its smaller size MFGs [6,45,66] leads to low butter yield compared to cow milk. Additionally, the high melting point of CM fat (41–42 °C) makes it difficult to churn the cream at the temperatures used for cow milk churning (8–12 °C) [102]. As a result, more force is needed to separate the fat globule membrane from the fat and to allow for the globules to adhere to each other, forming butter as water-in-oil emulsion [134]. The traditional churning method associated with higher churning force has recently been documented, resulting in less churning time and higher butterfat recovery from CM, compared to the common conventional mixing method [138]. Furthermore, it has a relatively low flavor content when compared to buffalo or cow milk, which would discourage consumer preferences [138]. The low flavor is mostly attributed to its low butyric acid content (less than 0.5%) compared to that in cow milk (about 5%) [11]. Moreover, it is difficult to distinguish butter color from other CM components during production, owing to its low β-carotene level [102,104]. The poor CM creaming production is also due to many other factors, such as the electrical charges on the globules, the ionic distribution, and the interfacial tension between milk serum and fat globules [55]. For this reason, producing CM butter by means of the same technique employed for cow milk butter may be difficult. As a result, the systematic use of already proven technology for cow milk butter is not always appropriate for CM butter, and adjustments based on more basic study on the behavior of milk fat components during processing are required. Moreover, transferring research results, which are now available, to an industrial scale is still insufficient, especially for the CM butter product, which requires additional technical and economic evaluations.

## 9. Conclusions and Future Prospects

The recent advances in understanding the composition of CM fats is important in understanding not only their nutritional value but also their functional properties and health benefits. The most abundant protein in the CM fat globule membrane (MFGM) was found to be lactadherin-like protein, which has anti-sticking and anti-bacterial qualities, and moreover it is suitable for consumers with an allergy to cow’s milk. The small size of the camel MFG resulted in its high-fat digestibility but led to difficulties in obtaining butter. CM has a low level of SFAs (C4-C6) and a high content of UFAs and LC–FAs. Hence, it contains an adequate number of components that are useful in reducing inflammation and decreasing the incidence of lipid-related cardiovascular diseases. The higher concentration of SFAs in CM fat is at the *sn*-2 position, whereas UFAs are primarily at the *sn*-1 and *sn*-3. CM has a higher concentration of total PLs than human and cow milk, and it may be used to substitute human milk since it delivers equal quantities of plasmalogens and SM to infants, even if the cholesterol content is a controversial issue. The main actual challenge is to better understand the properties of CM fat so that products with bioactive ingredients derived from milk can be designed and formulated.

## Figures and Tables

**Table 3:** Physicochemical properties of CM fat.

Type	Camel Milk		Cow Milk ^c^
	*C. dromedarius* ^a^	*C. bactrianus* ^b^	
Acid value	0.54	0.30–0.44	1.50
Refractive point	1.4490–1.4714	1.4563	1.4530
Saponification value	200.00–217.00	189.30–200.00	228.50
Iodine value	43.80–55.00	51.80–55.00	28.13–32.30
Polenske value	0.50–0.62	NS	1.56–1.61
Reichert–Meissel value	1.10–2.12	NS	28.40–29.56
Melting point (°C)	37.97–44.10	40.40–42.46	31.50–34.80

^a^ [89,102,106]. ^b^ [12,68]. ^c^ [89,107]. NS: not specified.

**Table 4:** Quantitative analysis of triacylglycerols in camel milk fat.

Studies	Samples	Extraction Method	Analysis Method	Major TAGs/Concentration
[20]	*C. dromedarius* (n = 5)	Folch	RP-HPLC/-APCI-MS	PPL (13.67%), POM (12.78%), PPO (11.77%), POO (10.67%), PPM (6.76%)
[91]	*C. dromedarius* (n = 20)	Folch	HPLC/ESI-MS	MPO (6.71%), PPO (5.72%), SPO (5.30%)
[14]	*C. dromedarius* (n = 20)	Rose–Gottlieb	GC	CN52 (20.37%), CN48 (21.60%), CN46 (12.48%), CN50 (25.79%)
[85]	*C. dromedarius*	Folch	UPLC/Q-TOF–MS	OPM/PPaP/PaMS (10.96%), OPaP/OMO/PLP (8.91%), OPP/OSM/PSPa (8.80%)
[12]	*C. dromedarius* and *C. bactrianus*	Mojonnier	UPC2/Q-TOF–MS	*C. dromedarius:* MPaPa/LMM/OMMy (5.36%), MPO (11.73%), SPP/MSS (7.15%), PaPaO (8.37%), PSS (7.88%) *C. bactrianus*: MMO/MyMS/LaPO (7.25%), MSP (8.52%), LaLS/MHH (7.04%), SPP/MSS (8.22%), PSS (5.91%)

Structure of fatty acids: M, C14:0; My, C14:1; P, C16:0; Pa, C16:1; L, C18:2; O, C18:1; S, C18:0; Reverse-phase high-performance liquid chromatography–atmospheric pressure chemical ionization mass spectrometr, (RP-HPLC/APCI-MS); Electrospray ionization-mass spectrometry, (ESI-MS); Gas chromatography, (GC); Ultra performance liquid chromatography–electrospray ionization–Quadrupole time-of-flight mass spectrometery, (UPLC/Q-TOF–MS); Ultra-performance convergence chromatography, (UPC^2^).

**Table 5:** Overview of camel milk phospholipid (PL) content and different analytical methods.

Studies	Samples	Extraction Method	Analysis Method	PLs Identified	Total PL Amounts
[124]	Israel, *C. dromedarius*	Folch	TLC	PI, PS, PE, PC, SM,	52.30%
[97]	Saudi Arabia, (n = 8, *C. dromedarius*)	Rose–Gottlieb	Ultroscan laser densitometer	ND	1.21%
[6]	Tunisia (n = 8, from 8 different *C. dromedarius*)	Folch	^31^P NMR	PE, PS, PI, PC, SM, aaPC, LPC	0.503 mmol/L
[20]	*C. bactrianus* (n = 20)	Folch	HPLC–ELSD	PI, PS, PE, PC, SM	4.65 mg/g fat
[128]	*C. dromedarius*	Folch	HPLC–UV	PI, PE, PS, PC	60–66 µg/mL
[110]	UAE, *C. dromedarius*	Folch	UPLC–ESI–QTOF–MS	PC, PE, PI, PS, SM, LPC	ND

United Arab Emirates, (UAE); phospholipids, (PLs); phosphatidylcholine, (PC); phosphatidylethanolamine, (PE); sphingomyelin, (SM); phosphatidylinositol, (PI); phosphatidylserine, (PS); lysophosphatidylcholine, (LPC); alkyl-acyl phosphatidylcholine, (aaPC); ND: not detected; Thin-layer chromatography, (TLC); High-performance liquid chromatography, (HPLC); ^31^P nuclear magnetic resonance, (^31^P NMR); Ultra-performance liquid chromatography electrospray ionization-quadrupole-time of flight-mass spectrometry, (UPLC-ESI-Q-TOF-MS).

## Data Availability

Not applicable.

## Abbreviations

CM	Camel milk
*C. dromedarius*	*Camelus dromedarius*
*C. bactrianus*	*Camelus bactrianus*
MFGs	Milk fat globules
MFGM	Milk fat globule membrane
FAs	Fatty acids
EFAs	Essential fatty acids
AA	Arachidonic acid
DHA	Docosahexaenoic acid
ALA	Alpha-linolenic acid
LA	Linoleic acid
EPA	Eicosapentaenoic acid
CLA	Conjugated linoleic acid
RA	Rumenic acid
LDL	Low-density lipoprotein
CN	Carbon number
TAGs	Triacylglycerols
SFAs	Saturated fatty acids
UFAs	Unsaturated fatty acids
MUFAs	Mono-unsaturated fatty acids
PUFAs	Poly-unsaturated fatty acids
SC-FAs	Short-chain fatty acids
MC-FAs	Medium-chain fatty acids
LC-FAs	Long-chain fatty acids
PLs	Phospholipids
PC	Phosphatidylcholine
PE	Phosphatidylethanolamine
SM	Sphingomyelin
PI	Phosphatidylinositol
PS	Phosphatidylserine

## References

[1] Khalesi M., Salami M., Moslehishad M., Winterburn J., Moosavi-Movahedi A.A. (2017). Biomolecular content of camel milk: A traditional superfood towards future healthcare industry. Trends Food Sci. Technol..

[2] Suliman G.M., Alowaimer A.N., Hussein E.O., Ali H.S., Abdelnour S.A., El-Hack M.E.A., Swelum A.A. (2019). Chemical Composition and Quality Characteristics of Meat in Three One-Humped Camel (*Camelus dromedarius*) Breeds as Affected by Muscle Type and Post-Mortem Storage Period. Animals.

[3] FAO (2019). Proceedings of the Gateway to Dairy Production and Products.

[4] Al-Sayyed H.F. (2020). Historical Background and Population of Camels. Handbook of Research on Health and Environmental Benefits of Camel Products.

[5] El-Agamy E.I. (2006). Camel milk. Handbook of Milk of Non-Bovine Mammals.

[6] Garcia C., Lutz N.W., Confort-Gouny S., Cozzone P.J., Armand M., Bernard M. (2012). Phospholipid fingerprints of milk from different mammalians determined by 31P NMR: Towards specific interest in human health. Food Chem..

[7] Konuspayeva G., Faye B. (2021). Recent Advances in Camel Milk Processing. Animals.

[8] Swelum A.A., El-Saadony M.T., Abdo M., Ombarak R.A., Hussein E.O.S., Suliman G., Alhimaidi A.R., Ammari A.A., Ba-Awadh H., Taha A.E. (2021). Nutritional, antimicrobial and medicinal properties of Camel’s milk: A review. Saudi J. Biol. Sci..

[9] Konuspayeva G.S. (2020). Camel milk composition and nutritional value. Handbook of Research on Health and Environmental Benefits of Camel Products.

[10] Meena S., Rajput Y., Sharma R. (2014). Comparative fat digestibility of goat, camel, cow and buffalo milk. Int. Dairy J..

[11] Konuspayeva G., Lemarie É., Faye B., Loiseau G., Montet D. (2008). Fatty acid and cholesterol composition of camel’s (Camelus bactrianus, *Camelus dromedarius* and hybrids) milk in Kazakhstan. Dairy Sci. Techn..

[12] Bakry I.A., Ali A.H., Abdeen E.-S., Ghazal A.F., Wei W., Wang X. (2020). Comparative characterisation of fat fractions extracted from Egyptian and Chinese camel milk. Int. Dairy J..

[13] He J., Xiao Y., Orgoldol K., Ming L., Yi L., Ji R. (2019). Effects of Geographic Region on the Composition of Bactrian Camel Milk in Mongolia. Animals.

[14] Smiddy M.A., Huppertz T., van Ruth S.M. (2012). Triacylglycerol and melting profiles of milk fat from several species. Int. Dairy J..

[15] Haddad I., Mozzon M., Strabbioli R., Frega N.G. (2010). Stereospecific analysis of triacylglycerols in camel (*Camelus dromedarius*) milk fat. Int. Dairy J..

[16] Bakry I.A., Korma S.A., Wei W., Nafea A.E., Mahdi A.A., Ziedan N.I., Wang X. (2021). Changes in the fatty acid content of Egyptian human milk across the lactation stages and in comparison with Chinese human milk. Eur. Food Res. Technol..

[17] Nyuar K., Min Y., Ghebremeskel K., Khalil A., Elbashir M., Cawford M. (2010). Milk of northern Sudanese mothers whose traditional diet is high in carbohydrate contains low docosahexaenoic acid. Acta Paediatr..

[18] Cardak A.D., Yetismeyen A., Bruckner H. (2003). Quantitative comparison of camel, goat and cow milk fatty acids. Milchwissenschaft.

[19] Maqsood S., Al-Dowaila A., Mudgil P., Kamal H., Jobe B., Hassan H.M. (2018). Comparative characterization of protein and lipid fractions from camel and cow milk, their functionality, antioxidant and antihypertensive properties upon simulated gastro-intestinal digestion. Food Chem..

[20] Zou X., Huang J., Jin Q., Guo Z., Liu Y., Cheong L.-Z., Xu X., Wang X. (2013). Lipid Composition Analysis of Milk Fats from Different Mammalian Species: Potential for Use as Human Milk Fat Substitutes. J. Agric. Food Chem..

[21] Nikkhah A. (2011). Science of Camel and Yak Milks: Human Nutrition and Health Perspectives. Food Nutr. Sci..

[22] Mann J. (2002). Diet and risk of coronary heart disease and type 2 diabetes. Lancet.

[23] Faye B., Bengoumi M., Al-Massaud A., Konuspayeva G. (2015). Comparative milk and serum cholesterol content in dairy cow and camel. J. King Saud Univ. Sci..

[24] Gorban A.M.S., Izzeldin O.M. (1999). Study on cholesteryl ester fatty acids in camel and cow milk lipid. Int. J. Food Sci. Technol..

[25] Alabdulkarim B. (2012). Effect of camel milk on blood glucose, cholesterol, triglyceride and liver enzymes activities in female albino rats. World Appl. Sci. J..

[26] Sulieman A.M.E., Elayan A.A., Saleh F. (2008). The Hypocholesterolemic Effect of Gariss and Gariss Containing Bifidobacteria in Rats Fed on a Cholesterol-Enriched Diet. Asian J. Biochem..

[27] Sboui A., Djegham M., Khorchani T., Hammadi M., Barhoumi K., Belhadj O. (2010). Effect of camel milk on blood glucose, cholesterol and total proteins variations in alloxan-induced diabetic dogs. Int. J. Diab. Metabol..

[28] Meena S., Rajput Y.S., Sharma R., Singh R. (2018). Effect of goat and camel milk vis a vis cow milk on cholesterol homeostasis in hypercholesterolemic rats. Small Rumin. Res..

[29] Karray N.L., Danthine S., Blecker C., Attia H. (2006). Contribution to the study of camel milk fat globule membrane. Int. J. Food Sci. Nutr..

[30] Simopoulos A.P. (2019). Genetic Variation and Evolutionary Aspects of Diet. Antioxidant Status, Diet, Nutrition and Health.

[31] Eaton S.B., Konner M. (1985). Paleolithic nutrition: A consideration of its nature and current implications. N. Engl. J. Med..

[32] Emken E.A. (1984). Nutrition and biochemistry of trans and positional fatty acid isomers in hydrogenated oils. Annu. Rev. Nutr..

[33] Shingfield K.J., Chilliard Y., Toivonen V., Kairenius P., Givens D.I. (2008). Trans Fatty Acids and Bioactive Lipids in Ruminant Milk. Bioact. Compon. Milk.

[34] Jenkins T., McGuire M. (2006). Major Advances in Nutrition: Impact on Milk Composition. Int. J. Dairy Sci..

[35] Dreiucker J., Vetter W. (2011). Fatty acids patterns in camel, moose, cow and human milk as determined with GC/MS after silver ion solid phase extraction. Food Chem..

[36] Teng F., Wang P., Yang L., Ma Y., Day L. (2017). Quantification of Fatty Acids in Human, Cow, Buffalo, Goat, Yak, and Camel Milk Using an Improved One-Step GC-FID Method. Food Anal. Methods.

[37] O’Shea M., Bassaganya-Riera J., Mohede I.C.M. (2004). Immunomodulatory properties of conjugated linoleic acid. Am. J. Clin. Nutr..

[38] Wang Y., Jones P.J.H. (2004). Dietary conjugated linoleic acid and body composition. Am. J. Clin. Nutr..

[39] Singh R., Mal G., Kumar D., Patil N.V., Pathak K.M.L. (2017). Camel Milk: An Important Natural Adjuvant. Agric. Res..

[40] Schrezenmeir J., Jagla A. (2000). Milk and diabetes. J. Am. Coll. Nutr..

[41] Liu C., Gelius E., Liu G., Steiner H., Dziarski R. (2000). Mammalian Peptidoglycan Recognition Protein Binds Peptidoglycan with High Affinity, Is Expressed in Neutrophils, and Inhibits Bacterial Growth. J. Biol. Chem..

[42] Redwan E.M., Tabll A. (2007). Camel Lactoferrin Markedly Inhibits Hepatitis C Virus Genotype 4 Infection of Human Peripheral Blood Leukocytes. J. Immunoass. Immunochem..

[43] Hamers-Casterman C., Atarhouch T., Muyldermans S., Robinson G., Hammers C., Songa E.B., Bendahman N. (1993). Naturally occurring antibodies devoid of light chains. Nature.

[44] Agrawal R., Beniwal R., Kochar D., Tuteja F., Ghorui S., Sahani M., Sharma S. (2005). Camel milk as an adjunct to insulin therapy improves long-term glycemic control and reduction in doses of insulin in patients with type-1 diabetes: A 1 year randomized controlled trial. Diabetes Res. Clin. Pr..

[45] Walter L., Shrestha P., Fry R., Leury B., Logan A. (2020). Lipid metabolic differences in cows producing small or large milk fat globules: Fatty acid origin and degree of saturation. J. Dairy Sci..

[46] Månsson H.L. (2008). Fatty acids in bovine milk fat. Food Nutr. Res..

[47] Yang Y., Zheng N., Zhao X., Zhang Y., Han R., Ma L., Zhao S., Li S., Guo T., Wang J. (2015). Proteomic characterization and comparison of mammalian milk fat globule proteomes by iTRAQ analysis. J. Proteom..

[48] Sabha B.H., Masood A., Alanazi I.O., Alfadda A.A., Almehdar H.A., Benabdelkamel H., Redwan E.M. (2020). Comparative Analysis of Milk Fat Globular Membrane (MFGM) Proteome between Saudi Arabia Camelus dromedary Safra and Wadha Breeds. Molecules.

[49] Saadaoui B., Henry C., Khorchani T., Mars M., Martin P., Cebo C. (2013). Proteomics of the milk fat globule membrane from C amelus dromedarius. Proteomics.

[50] Evers J.M. (2004). The milkfat globule membrane—compositional and structural changes post secretion by the mammary secretory cell. Int. Dairy J..

[51] Lopez C. (2011). Milk fat globules enveloped by their biological membrane: Unique colloidal assemblies with a specific composition and structure. Curr. Opin. Colloid Interface Sci..

[52] Lee H., Padhi E., Hasegawa Y., Larke J., Parenti M., Wang A., Hernell O., Lönnerdal B., Slupsky C. (2018). Compositional Dynamics of the Milk Fat Globule and Its Role in Infant Development. Front. Pediatr..

[53] Keenan T.W. (2001). Historical Perspective: Milk Lipid Globules and Their Surrounding Membrane: A Brief History and Perspectives for Future Research. J. Mammary Gland. Biol. Neoplasia.

[54] Attia H., Kherouatou N., Fakhfakh N., Khorchani T., Trigui N. (2000). Dromedary Milk Fat: Biochemical, Microscopic and Rheological Characteristics. J. Food Lipids.

[55] Farah Z., Rüegg M. (1991). The Creaming Properties and Size Distribution of Fat Globules in Camel Milk. J. Dairy Sci..

[56] Attaie R., Richter R. (2000). Size Distribution of Fat Globules in Goat Milk. J. Dairy Sci..

[57] Manoni M., Di Lorenzo C., Ottoboni M., Tretola M., Pinotti L. (2020). Comparative Proteomics of Milk Fat Globule Membrane (MFGM) Proteome across Species and Lactation Stages and the Potentials of MFGM Fractions in Infant Formula Preparation. Foods.

[58] Bauer E., Jakob S., Mosenthin R. (2005). Principles of Physiology of Lipid Digestion. Asian-Australas. J. Anim. Sci..

[59] El Fakharany E., El-Baky N.A., Linjawi M.H., AlJaddawi A.A., Saleem T.H., Nassar A.Y., Osman A., Redwan E.M. (2017). Influence of camel milk on the hepatitis C virus burden of infected patients. Exp. Ther. Med..

[60] Imam A., Drushella M.M., Taylor C.R., Tökés Z.A. (1986). Preferential expression of a Mr 155,000 milk-fat-globule membrane glycoprotein on luminal epithelium of lobules in human breast. Cancer Res..

[61] Khatoon H., Ikram R., Anser H., Naeem S., Khan S.S., Fatima S., Sultana N., Sarfaraz S. (2019). Investigation of anti-inflammatory and analgesic activities of camel milk in animal models. Pak. J. Pharm. Sci..

[62] Mulder H., Walstra P. (1974). The Milk Fat Globule.

[63] Heid H.W., Keenan T.W. (2005). Intracellular origin and secretion of milk fat globules. Eur. J. Cell Biol..

[64] Robenek H., Buers I., Hofnagel O., Robenek M.J., Troyer D., Severs N.J. (2009). Compartmentalization of proteins in lipid droplet biogenesis. Biochim. Biophys. Acta (BBA)—Mol. Cell Biol. Lipids.

[65] Masedunskas A., Chen Y., Stussman R., Weigert R., Mather I.H. (2017). Kinetics of milk lipid droplet transport, growth, and secretion revealed by intravital imaging: Lipid droplet release is intermittently stimulated by oxytocin. Mol. Biol. Cell.

[66] El-Zeini H.M. (2006). Microstructure, rheological and geometrical properties of fat globules of milk from different animal species. Polish J. Food Nutr. Sci..

[67] Habtegebriel H., Wawire M., Gaukel V., Taboada M.L. (2020). Comparison of the viscosity of camel milk with model milk systems in relation to their atomization properties. J. Food Sci..

[68] Zhao D.B. (2006). Studies on the Chemical Composition and Chemical–Physical Properties of Alxa Bactrian Camels Milk in Inner Mongolia. Master’s Thesis.

[69] Berton A., Rouvellac S., Robert B., Rousseau F., Lopez C., Crenon I. (2012). Effect of the size and interface composition of milk fat globules on their in vitro digestion by the human pancreatic lipase: Native versus homogenized milk fat globules. Food Hydrocoll..

[70] Favé G., Coste T., Armand M. (2004). Physicochemical properties of lipids: New strategies to manage fatty acid bioavailability. Cell. Mol. Boil..

[71] D’Urso S., Cutrignelli M.I., Calabrò S., Bovera F., Tudisco R., Piccolo V., Infascelli F. (2008). Influence of pasture on fatty acid profile of goat milk. J. Anim. Physiol. Anim. Nutr..

[72] Briard V., Leconte N., Michel F., Michalski M.-C. (2003). The fatty acid composition of small and large naturally occurring milk fat globules. Eur. J. Lipid Sci. Technol..

[73] Fauquant C., Leconte N., Michalski M.-C. (2005). Differently sized native milk fat globules separated by microfiltration: Fatty acid composition of the milk fat globule membrane and triglyceride core. Eur. J. Lipid Sci. Technol..

[74] Aoki N., Ishii T., Ohira S., Yamaguchi Y., Negi M., Adachi T., Nakamura R., Matsuda T. (1997). Stage specific expression of milk fat globule membrane glycoproteins in mouse mammary gland: Comparison of MFG-E8, butyrophilin, and CD36 with a major milk protein, β-casein. Biochim. Biophys. Acta (BBA)—Gen. Subj..

[75] Jenness R. (1980). Composition and Characteristics of Goat Milk: Review 1968−1979. Int. J. Dairy Sci..

[76] Zouari A., Schuck P., Gaucheron F., Triki M., Delaplace G., Gauzelin-Gaiani C., Lopez C., Attia H., Ayadi M.A. (2019). Microstructure and chemical composition of camel and cow milk powders’ surface. LWT.

[77] Konuspayeva G., Faye B., Loiseau G. (2009). The composition of camel milk: A meta-analysis of the literature data. J. Food Compos. Anal..

[78] Ohlsson L. (2010). Dairy products and plasma cholesterol levels. Food Nutr. Res..

[79] Saini R.K., Keum Y.-S. (2018). Omega-3 and omega-6 polyunsaturated fatty acids: Dietary sources, metabolism, and significance—A review. Life Sci..

[80] Virtanen J.K., Mursu J., Tuomainen T.-P., Voutilainen S. (2014). Dietary fatty acids and risk of coronary heart disease in men: The Kuopio Ischemic Heart Disease Risk Factor Study. Arterioscler. Thromb. Vasc. Biol..

[81] Karray N., Lopez C., Ollivon M., Attia H. (2005). La matière grasse du lait de dromadaire: Composition, microstructure et polymorphisme. Une revue. Oléagineux Corps Gras Lipides.

[82] Yang J., Zheng N., Wang J., Yang Y. (2017). Comparative milk fatty acid analysis of different dairy species. Int. J. Dairy Technol..

[83] Zhang H., Yao J., Zhao D., Liu H., Li J., Guo M. (2005). Changes in Chemical Composition of Alxa Bactrian Camel Milk During Lactation. J. Dairy Sci..

[84] Yi L., Te M.L., Zheng Z.Q., Ming L., Er D.M.T., Chen G.L., Ji R.M.T. (2014). Study on seasonal variation of fatty acid composition of Bactrian camel’s milk in Zhunger. Dairy Ind. China.

[85] Ali A.H., El-Wahed E.M.A., Abed S.M., Korma S.A., Wei W., Wang X. (2019). Analysis of triacylglycerols molecular species composition, total fatty acids, and sn-2 fatty acids positional distribution in different types of milk powders. J. Food Meas. Charact..

[86] Konuspayeva G., Faye B., Mussaad A. (2014). Some lipid components of the camel milk and blood in intensive farm insaudi arabia. Emir. J. Food Agric..

[87] Ereifej K.I., Alu’Datt M.H., AlKhalidy H.A., Alli I., Rababah T. (2011). Comparison and characterisation of fat and protein composition for camel milk from eight Jordanian locations. Food Chem..

[88] Ayadi M., Hammadi M., Casals R., Atigui M., Khorchani T., Samara E.M., Abdoun K.A., Al-Haidary A.A., Caja G. (2018). Influence of management type and stage of lactation on the performance and milk fatty acid profile of dairy camels (*Camelus dromedaries*). J. Agric. Sci..

[89] Abu-Lehia I.H. (1989). Physical and chemical characteristics of camel milkfat and its fractions. Food Chem..

[90] Mohamed E., Mustafa A. (2013). Fatty Acids Content in Milk of Dromedary Camel (*Camelus dromedarius*) from Farming and Pastoral Systems in Sudan. Inter J. Sci. Res..

[91] Haddad I., Mozzon M., Strabbioli R., Frega N.G. (2011). Electrospray ionization tandem mass spectrometry analysis of triacylglycerols molecular species in camel milk (*Camelus dromedarius*). Int. Dairy J..

[92] Chamekh L., Calvo M., Khorchani T., Castro-Gómez P., Hammadi M., Fontecha J., Yahyaoui M.H., Latifa C., Marivi C., Touhami K. (2020). Impact of management system and lactation stage on fatty acid composition of camel milk. J. Food Compos. Anal..

[93] Pérez-Jiménez F., Castro P., López-Miranda J., Paz-Rojas E., Blanco A., López-Segura F., Velasco F., Marin C., Fuentes F., Ordovás J.M. (1999). Circulating levels of endothelial function are modulated by dietary monounsaturated fat. Atherosclerosis.

[94] Wei W., Jin Q., Wang X. (2019). Human milk fat substitutes: Past achievements and current trends. Prog. Lipid Res..

[95] Burlingame B., Nishida C., Uauy R., Weisell R. (2009). Fats and Fatty Acids in Human Nutrition: Introduction. Ann. Nutr. Metab..

[96] Wongtangtintharn S., Oku H., Iwasaki H., Toda T. (2004). Effect of branched-chain fatty acids on fatty acid biosynthesis of human breast cancer cells. J. Nutr. Sci. Vitaminol..

[97] MS Gorban A., Izzeldin O.M. (2001). Fatty acids and lipids of camel milk and colostrum. Int. J. Food Sci. Nutr..

[98] Dowelmadina I.M.M., El Zubeir I.E.M., Arabi O., Abakar A.D. (2019). Omega-3 fatty acids in milk fat of some Sudanese camels. J. Dairy Res.Technol..

[99] Faye B., Konuspayeva G., Narmuratova Z., Serikbaeva A., Musaad A.M., Mehri H. (2013). Effect of crude olive cake supplementation on camel milk production and fatty acid composition. Dairy Sci. Technol..

[100] Leparmarai P.T., Kunz C., Mwangi D.M., Gluecks I., Kreuzer M., Marquardt S. (2021). Camels and cattle respond differently in milk phenol excretion and milk fatty acid profile to free ranging conditions in East-African rangelands. Sci. Afr..

[101] Koletzko B., Bergmann K., Brenna J.T., Calder P.C., Campoy C., Clandinin M.T., Colombo J., Daly M., Decsi T., Demmelmair H. (2019). Should formula for infants provide arachidonic acid along with DHA? A position paper of the European Academy of Paediatrics and the Child Health Foundation. Am. J. Clin. Nutr..

[102] Farah Z., Streiff T., Bachmann M. (1989). Manufacture and characterization of camel milk butter. Milchwissenschaft.

[103] Zhao D.-B., Bai Y.-H., Niu Y.-W. (2015). Composition and characteristics of Chinese Bactrian camel milk. Small Rumin. Res..

[104] Abu-Lehia I.H. (1987). Composition of camel milk. Milchwissenschaft.

[105] Buldo P. (2013). Crystallization of Fat in and Outside Milk Fat Globules—Effect of Processing and Storage Conditions. Ph.D. Thesis.

[106] Purchase H.S. (1943). Some Experiments in the Making of Butter, Ghee, and Cheese from Camels’ Milk. East. Afr. Agric. J..

[107] Bharwade M., Balakrishnan S., Chaudhary N., Jain A. (2017). Fatty Acid Profile and Physico-Chemical Characteristics of Milk Lipids of Kankrej Cow. Int. J. Curr. Microbiol. Appl. Sci..

[108] Wright A.J., Marangoni A.G. (2006). Crystallization and Rheological Properties of Milk Fat. Advanced Dairy Chem Volume 2 Lipids.

[109] Hansen H.S., Jensen B. (1985). Essential function of linoleic acid esterified in acylglucosylceramide and acylceramide in maintaining the epidermal water permeability barrier. Evidence from feeding studies with oleate, linoleate, arachidonate, columbinate and α-linolenate. Biochim. Biophys. Acta (BBA)—Lipids Lipid Metab..

[110] Ali A., Zou X., Huang J., Abed S.M., Tao G., Jin Q., Wang X. (2017). Profiling of phospholipids molecular species from different mammalian milk powders by using ultra-performance liquid chromatography-electrospray ionization-quadrupole-time of flight-mass spectrometry. J. Food Compos. Anal..

[111] Jenkins B., West J.A., Koulman A. (2015). A Review of Odd-Chain Fatty Acid Metabolism and the Role of Pentadecanoic Acid (C15:0) and Heptadecanoic Acid (C17:0) in Health and Disease. Molecules.

[112] Gresti J., Bugaut M., Maniongui C., Bezard J. (1993). Composition of Molecular Species of Triacylglycerols in Bovine Milk Fat. J. Dairy Sci..

[113] Bornaz S., Fanni J., Parmentier M. (1993). Butter texture: The prevalent triglycerides. J. Am. Oil Chem. Soc..

[114] Miles E.A., Calder P.C. (2017). The influence of the position of palmitate in infant formula triacylglycerols on health outcomes. Nutr. Res..

[115] Bar-Yoseph F., Lifshitz Y., Cohen T. (2013). Review of sn-2 palmitate oil implications for infant health. Prostaglandins Leukot. Essent. Fat. Acids.

[116] Farag S.I., Kebary K.M.K. (1992). Chemical composition and physical properties of camel’s milk and milk fat. Proceedings of the 5th Egyptian Conference for Dairy Science and Technology.

[117] Parodi P.W. (2009). Has the association between saturated fatty acids, serum cholesterol and coronary heart disease been over emphasized?. Int. Dairy J..

[118] Wasfi I., Hafez A., El Tayeb F., El Taher A. (1987). Thyroid hormones, cholesterol and triglyceride levels in the camel. Res. Veter Sci..

[119] Kamal A.M., Salama O.A. (2009). Lipid Fractions and Fatty Acid Composition of Colostrums, Transitional and Mature She-Camel Milk During the First Month of Lactation. Asian J. Clin. Nutr..

[120] Barłowska J., Szwajkowska M., Litwińczuk Z., Król J. (2011). Nutritional Value and Technological Suitability of Milk from Various Animal Species Used for Dairy Production. Compr. Rev. Food Sci. Food Saf..

[121] Li H., Papadopoulos V. (1998). Peripheral-Type Benzodiazepine Receptor Function in Cholesterol Transport. Identification of a Putative Cholesterol Recognition/Interaction Amino Acid Sequence and Consensus Pattern. Endocrinology.

[122] Buonopane G.J., Kilara A., Smith J.S., McCarthy R.D. (1992). Effect of skim milk supplementation on blood cholesterol concentration, blood pressure, and triglycerides in a free-living human population. J. Am. Coll. Nutr..

[123] Rao D.R., Chawan C.B., Pulusani S.R. (1981). Influence of Milk and Thermophilus Milk on Plasma Cholesterol Levels and Hepatic Cholesterogenesis in Rats. J. Food Sci..

[124] Morrison W.R. (1968). The distribution of phospholipids in some mammalian milks. Lipids.

[125] Tanaka K., Hosozawa M., Kudo N., Yoshikawa N., Hisata K., Shoji H., Shinohara K., Shimizu T. (2013). The pilot study: Sphingomyelin-fortified milk has a positive association with the neurobehavioural development of very low birth weight infants during infancy, randomized control trial. Brain Dev..

[126] Nishimukai M., Wakisaka T., Hara H. (2003). Ingestion of plasmalogen markedly increased plasmalogen levels of blood plasma in rats. Lipids.

[127] Helmerich G., Koehler P. (2003). Comparison of Methods for the Quantitative Determination of Phospholipids in Lecithins and Flour Improvers. J. Agric. Food Chem..

[128] Yassin A.M., Hamid M.I.A., Farid O.A., Amer H., Warda M. (2016). Dromedary milk exosomes as mammary transcriptome nano-vehicle: Their isolation, vesicular and phospholipidomic characterizations. J. Adv. Res..

[129] Shirouchi B., Nagao K., Furuya K., Inoue N., Inafuku M., Nasu M., Otsubo K., Koga S., Matsumoto H., Yanagita T. (2009). Effect of dietary phosphatidylinositol on cholesterol metabolism in Zucker (fa/fa) rats. J. Oleo Sci..

[130] Mukherjee S., Maxfield F.R. (2004). Membrane domains. Annu. Rev. Cell Dev. Biol..

[131] Yavin E., Brand A., Green P. (2002). Docosahexaenoic Acid Abundance in the Brain: A biodevice to Combat Oxidative Stress. Nutr. Neurosci..

[132] Labadaridis I., Moraitou M., Theodoraki M., Triantafyllidis G., Sarafidou J., Michelakakis H. (2009). Plasmalogen levels in full-term neonates. Acta Paediatr..

[133] Mourad K., Nour-Eddine K. (2006). Physicochemical and microbiological study of “shmen”, a traditional butter made from camel milk in the Sahara (Algeria): Isolation and identification of lactic acid bacteria and yeasts. Grasas Aceites.

[134] Yagil R. (1982). Camels and Camel Milk: FAO Animal Production and Health.

[135] Parmar N.B. (2013). Characterization of Ghee Prepared from Camel Milk and Evaluation of Its Shelf Life During Storage. Ph.D. Thesis.

[136] Knoess K.H., Makhudum A.J., Rafiq M., Hafeez M. (1986). Milk production potential of the dromedary with special reference to the province of Punjab, Pakistan. World Anim. Rev..

[137] Mtibaa I., Zouari A., Attia H., Ayadi M.A., Danthine S. (2021). Effects of Physical Ripening Conditions and Churning Temperature on the Butter-Making Process and the Physical Characteristics of Camel Milk Butter. Food Bioprocess. Technol..

[138] Berhe T., Seifu E., Kurtu M.Y. (2013). Physicochemical properties of butter made from camel milk. Int. Dairy J..

[139] Bylund G. (1995). Dairy processing handbook: Tetra Pak Processing Systems AB.

[140] Khan K.U., Appanna T.C. (1967). Carotene and vitamin A in camel milk. Indian J. Nutr. Diet..

